# Frequency and Antimicrobial Resistance Patterns of Bacterial Species Isolated from the Body Surface of the Housefly (*Musca domestica*) in Akure, Ondo State, Nigeria

**DOI:** 10.18502/jad.v14i1.2715

**Published:** 2020-03-31

**Authors:** Babatunde Odetoyin, Babatunde Adeola, Olarinde Olaniran

**Affiliations:** 1 Department of Medical Microbiology and Parasitology, College of Health Sciences, Obafemi Awolowo University, Ile-Ife, Osun State, Nigeria; 2 Department of Microbiology, Federal University of Technology, Akure, Ondo State, Nigeria

**Keywords:** Houseflies, Antimicrobial resistance, Pathogenic bacteria, Vector, Infection

## Abstract

**Background:**

The emergence and spread of antibiotic resistant bacteria has become a serious problem worldwide. Houseflies are potential carriers of pathogenic and resistant bacteria and could be contributing to the global spread of these strains in the environments. We investigated the prevalence and antimicrobial resistant profiles of bacteria isolated from houseflies in Akure.

**Methods:**

Twenty-five houseflies were captured by a sterile nylon net from the slaughterhouse, garbage dump, human house, hospital, and eatery from 9:00am to 1:00pm when the flies were active and transported immediately to the laboratory in sterile containers for processing. Bacterial loads were enumerated by serial dilution and plating on nutrient agar and selective media. Bacteria species were isolated by conventional isolation technique. Antibiotic susceptibility test was determined by the Kirby-Bauer disc diffusion technique.

**Results:**

Sixty-seven bacterial species were isolated from 25 samples that were collected. The predominant bacterial species was *Escherichia coli* (n= 31, 45%), followed by *Klebsiella pneumoniae* (n= 17, 25%), *Staphylococcus aureus* (n= 11, 16%) and *Pseudomonas aeruginosa* (n= 3, 4.3%). The bacterial load of the samples ranged from 9.7×10^5^CFU/mL to 1.65×10^6^CFU/mL*.* The results revealed that all isolates of *Pseudomonas aeruginosa*, *Salmonella* spp, and *Proteus mirabilis* were resistant to streptomycin and cotrimoxazole, augmentin and amoxicillin respectively. None of the *S. aureus* isolates was resistant to cotrimoxazole, chloramphenicol, sparfloxacin, augmentin, and ofloxacin. All isolates were multi-drug resistant.

**Conclusion:**

House flies that were collected from the slaughterhouse, garbage dump, human house, hospital, and eatery may participate in the dispersal of pathogenic and resistant bacteria in the study environment.

## Introduction

The emergence of antimicrobial resistance in bacteria has become a serious global problem (1). A WHO report indicates that this problem is contributing to the increase in the cost of diagnosing and treating resistant infections (2). Today, the main concern is that antimicrobial production pipelines are drying up and very few available therapeutic options are effective for common infections.

Antimicrobial resistant bacteria are usually spread from person to person, or from the non-human sources in the environment. Available data indicate that the house fly, a known cosmopolitan pest with a worldwide distribution and commonly found in close association with human activities could also contribute to the global spread of pathogenic as well as resistant bacteria in the environments (3–5). Houseflies are known to act as mechanical vectors of pathogenic bacteria like *Vibrio cholerae*, *Escherichia coli*, *Salmonella* and *Shigella* (6–8). They pick up the pathogens on their bodies with the aid of their hairy proboscis and feet and disseminate them by regurgitating vomits and depositing faecal droplets during the feeding process. These vectors have also been reported to be carriers of multi-drug resistant bacteria in hospital environments, and they may participate in the spread of resistant as well as pathogenic pathogens within hospitals (9). Adequate control of these vectors would allow a reduction of the transmission of these pathogenic and resistant bacteria. In spite of the awareness of the dangers posed by these flies and their link with poor environmental sanitation, factors/practices such as indiscriminate refuse dumping and waste disposal, bad drainage systems coupled with improper handling of food still abound in Nigeria. Since pathogenic microorganisms are widespread in our environment, there is abundant opportunity for flies to become contaminated and, in turn, contaminate the environment. Hence, the aim of the study was to isolate and characterize the bacterial pathogens on the external surfaces of houseflies and determine the antibiotic resistance patterns of the isolated organism to commonly used antimicrobials. The data from this study will provide information on the dangers posed by these flies in the environment. Effective communication of the information is expected to generate apposite ideas for acceptable and workable interventions to control the spread of resistant pathogens in our environment.

## Materials and Methods

### Study location, sample collection and processing

The study was carried out in Akure, the capital of Ondo state which is located in the South-western part of Nigeria. Akure lies on latitude 7°15′North of the Equator and on longitude 5°15′east of the Greenwich meridian (10).

Twenty-five samples of houseflies were captured by sterile nylon nets from five different locations. Flies were caught from the selected sites (slaughterhouse, garbage dump, human house, hospital, and eatery) during the period of study with sterile nets from 9:00am to 1:00 pm when the flies were active. The collected flies were placed in sterile vials and transported to the laboratory for identification by an entomologist. All species other than *Musca domestica* were removed. After identification, 2ml of sterile normal saline solution was added to each vial that contained the fly and shaken vigorously for one minute. The fly was removed from the saline and was checked for bacteria dislodged from the external surface of the fly (6).

### Bacterial counts and Isolation of microorganisms

The diluents used for the samples were the sterile saline solution. One ml was taken from each sample using a sterile syringe and added to nine ml of sterile distilled water in the test tubes. This dilution process was repeated until the 4th dilution was obtained. From the serially diluted samples, 1ml each of the 10^−4^ dilutions of the housefly samples was taken aseptically with the use of a sterile syringe, and pour-plated on the nutrient agar plates, and then incubated at 37 °C for 24 hours. The colonies on the plates were then counted and their morphological features recorded (11). From the nutrient agar plate, sterile inoculating loop previously flamed to red-hot and cooled was used to pick different colonies from all the isolation plates and then streaked on nutrient agar and incubated at 37 °C for 24 hours; the morphological features of the distinct colonies along the line of streak were observed and used to infer the type of organisms present on the isolation plates (12). The biochemical characterization of isolates was carried out as described by Cheesbrough (13).

### Antibiotic sensitivity test

The antimicrobial susceptibility patterns of the isolates were determined by the Kirby-Bauer disc diffusion technique on Mueller-Hinton agar (CM0337) (Oxoid Ltd., Basingstoke, Hampshire, England). Antibiotics tested were augmentin (30μg), ofloxacin (10μg), chloramphenicol (30μg), gentamicin (10μg), (10μg), sparfloxacin (10μg), amoxicillin (25μg), ciprofloxacin (10μg), streptomycin (30μg), pefloxacin (5μg) and cotrimoxazole (30μg) (Remel, USA). The plates were incubated at 37 °C for 24 hours. The diameters of the zones of inhibition were measured with a ruler and interpreted according to the guidelines of the Clinical and Laboratory Standard Institute (CLSI) (14).

### Statistical Analysis

Data were presented as frequencies and percentages. Independent T-test and analysis of variance test of SPSS version 20 software package (SPSS, Inc. Chicago, Illinois) were used to determine the significance of the data. All p-values were two-sided and a p-value that was less than or equal to 0.05 was considered to be statistically significant.

## Results

### Microbiological analysis

The results of the microbiological analysis showed that total bacterial counts ranged from 9.68×10^5^ CFU/mL to 1.65×10^6^ CFU/mL. The highest load of bacteria was found in samples from hospital (1.65×10^6^ CFU/mL), followed by eatery (1.60×10^6^ CFU/mL), garbage dump (1.59X10^6^ CFU/mL) and human house (1.28×10^6^ CFU/mL). There was no significant difference in the mean load of bacteria from the five sampled sites (F= 2.7836, p= 0.0547). However, the mean load of bacteria from hospital was significantly higher (1.276×10^6^ CFU/mL) than the mean load of bacteria from slaughterhouse (9.68 ×10^5^ CFU/mL) (t= −2.79503, p= 0.0233) (Table 1).

**Table 1.** Bacterial load of sample (CFU/mL) (10^4^)^a^

**Colony**	**Slaughterhouse**	**Human House**	**Hospital**	**Eatery**	**Garbage Dump**
**Plate 1**	86	84	102	184	206
**Plate 2**	102	184	158	146	178
**Plate 3**	56	98	196	96	164
**Plate 4**	84	128	204	206	142
**Plate 5**	156	144	166	168	108
**Average**	96.8^b^	127.6	165.2^b^	160	159.6

A = F= 2.7836, p= 0.054752;

B = t= −2.79503, p= 0.0233

### Organisms isolated from different samples

Sixty-seven bacterial species were isolated from the external surfaces of 25 identified house flies. These were divided into six genera comprising *Klebsiella*, *Staphylococcus*, *Escherichia*, *Pseudomonas*, *Proteus*, and *Salmonella* (Table 2). The commonest bacterial species identified was *E. coli* (n= 31, 46.3%), followed by *Klebsiella pneumoniae* (n= 17, 25.4%), *Staphylococcus aureus* (n= 11, 16.4%) and *Pseudomonas aeruginosa* (n= 3; 4.5%).

**Table 2.** Number of isolates from all sample locations

**Isolates**	**Sample locations**					

	**Slaughter house (n= 12)**	**Human House (n= 15)**	**Hospital (n= 14)**	**Eatery (n= 11)**	**Garbage Dump (15)**	**Total (67)**
***Klebsiella pneumoniae***	0 (0)	4(26.7)	7(50)	2(18.2)	4(26.7)	17(25.4)
***Escherichia coli***	6 (50)	8(53.3)	2(14.3)	6(54.5)	9(60)	31(46.3)
***Staphylococcus aureus***	3(25)	3(20)	3(21.4)	0 (0)	2(13.3)	11(16.4)
***Pseudomonas aeruginosa***	1(8.3)	0 (0)	2(14.3)	0 (0)	0 (0)	3(4.5)
***Proteus* spp**	2 (16.7)	0 (0)	0 (0)	0 (0)	0 (0)	2(3)
***Salmonella* spp**	0 (0)	0 (0)	0 (0)	3(27.3)	0 (0)	3(4.5)

Of all the different sites sampled, garbage dumps and human houses harboured the highest number of bacteria species (n= 15). *Escherichia coli* was the commonest bacterial species isolated in the slaughterhouse (n= 3), human house (n= 8), eatery (n= 6) and garbage dump (n= 9). However, *K. pneumoniae* was the commonest bacterial species isolated from the hospital (n= 7).

### Baseline resistance rates of isolated bacteria

All isolates (100%) of *P. aeruginosa*, *Salmonella* spp, and *Proteus* spp were resistant to streptomycin, cotrimoxazole, augmentin, and amoxicillin. No isolate of *S. aureus* was resistant to chloramphenicol, sparfloxacin, augmentin, and ofloxacin. Isolates of *E. coli* were commonly resistant to augmentin (n= 28, 90.3%) and cotrimoxazole (n= 20, 64.5%). The Least resistance rates were exhibited by *K. pneumoniae* (n= 1, 5.9%), *E. coli* (n= 1, 3.2%), and *S. aureus* (n= 1, 9.1%) to ciprofloxacin (Table 3).

**Table 3.** Antimicrobial resistant pattern of identified bacteria in all sample locations

**Isolates**	**Antimicrobial agents**									

	**PER**	**GEN**	**AMO**	**CPX**	**S**	**SXT**	**CH**	**SP**	**AU**	**OFX**
***Staphylococcus aureus* (n= 11)**	2 (18.2)	3 (27.3)	7 (63.6)	1 (9.1)	3 (27.3)	5 (45.5)	0 (0)	0 (0)	0 (0)	0 (0)
***Salmonella* spp (n= 3)**	1 (33.3)	0 (0)	1 (33.3)	1 (33.3)	3 (100)	1 (33.3)	1 (33.3)	0 (0)	3 (100)	1 (33.3)
***Proteus* spp (n= 2)**	1 (50)	1 (50)	2 (100)	1 (50)	2 (100)	2 (100)	1 (50)	1 (50)	0 (0)	2 (100)
***Klebsiella pneumonia* (n=17)**	4 (23.5)	5 (29.4)	8 (47.1)	1 (5.9)	9 (52.9)	2 (11.8)	1 (5.9)	2 (11.8)	13 (76.4)	11 (64.7)
***Escherichia coli* (n= 31)**	2 (6.5)	3 (9.7)	18 (58.1)	1 (3.2)	23 (74.2)	20 (64.5)	4 (12.9)	2 (6.5)	28 (90.3)	5 (16.1)
***Pseudomonas aeruginosa* (n= 3)**	2 (66.7)	1 (33.3)	1 (33.3)	1 (33.3)	3 (100)	3 (100)	1 (33.3)	1 (33.3)	0 (0)	1 (33.3)

PER= Perfloxacin (PER 30μg), GEN= Gentamycin (GEN 30μg), AMO= Amoxicillin (AMO 30μg), CPX= Ciprofloxacin (CPX), S= Streptomycin (S 30μg), SXT= Cotrimoxazole (SXT 30μg), CH= Chloramphenicol (CH 30μg), SP= Sparfloxacin (SP 10μg), AU= Augmentin (AU 30μg), and OFX= Ofloxacin (OFX 10μg)

**Fig. 1.**
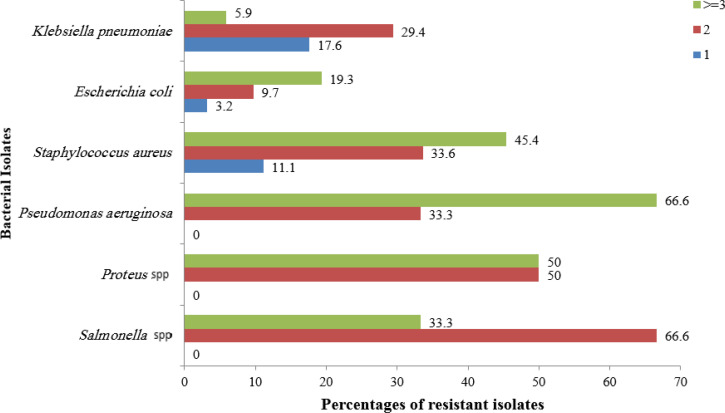
Multidrug Resistance patterns of isolates

Multidrug resistance was defined as the resistance of isolates to at least one antibiotic in three or more classes of antibiotics. As shown in (Fig. 1), all isolates were multidrug-resistant, with 66.6% of isolates of *P. aeruginosa*, 45.4% of *S. aureus*, 33.3% of *Salmonella* spp., 19.3% of *E. coli* strains and 5.9% of *K. pneumoniae* resistant to three or more classes of antibiotics.

## Discussion

In the present study, we investigated the frequency and antimicrobial resistant profiles of bacteria isolated from houseflies in five different locations (slaughterhouse, garbage dump, human house, hospital, and eatery). Six bacterial species, comprising *Salmonella*, *Proteus*, *S. aureus*, *P. aeruginosa*, *E. coli*, and *K. pneumoniae* were isolated from *M. domestica.* Similar findings have also been reported by previous investigators who have highlighted the importance of houseflies as mechanical vectors of various pathogens (15–17). The external organs of *M. domestica* constitute a large source of bacteria, and their persistent association with humans, animals, food, refuse, and excreta makes them potential mechanical or biological vectors for the dissemination of pathogenic and multidrug-resistant bacteria (18–22). The results of this study indicate that *M. domestica* plays a great role as a mechanical carrier of bacteria in this environment, most of the bacteria isolated which have also been isolated by previous investigators are of medical importance (17, 23, 24).

Of all the different sites sampled, garbage dump and human house harboured the highest number of bacterial species (n= 15). Garbage dumps are sites where waste products are kept, and they serve as media for breeding microbes. Also, these dumpsites serve as breeding sites for flies which while feeding could also convey microbes from one place to another thereby affecting the health of the community. Apart from garbage dumps, we also observed the highest number of bacteria from human house and hospital which may be due to improper waste disposal and environmental sanitation. Our finding is in tandem with the study of Nazari et al. (25) that also reported in large numbers the isolation of bacteria from hospital and non-hospital environments which they attributed to a low level of general hygiene.

*Escherichia coli* was the commonest bacterial sp isolated in the slaughterhouse (n= 3), human house (n= 8), eatery (n= 6) and garbage dump (n= 9). However, *K. pneumoniae* was the commonest bacteria sp isolated from the hospital (n= 7). *Klebsiella* spp and *E. coli* are gram-negative organisms that occupy a variety of niches like other members of enterobacteriaceae. Their isolation from nearly all sources may not be unconnected with their ubiquity, and with the fact that house flies feed mainly on feaces and other animal waste, which is a rich source of enteric bacteria (26). *Escherichia coli* is known to cause diarrhoea including traveller’s diarrhoea, and haemolytic uremic syndrome, which people can contract by eating contaminated food. Isolation of this pathogen from the eatery, the slaughterhouse, the human house could lead to outbreaks of *E. coli* gastroenteritis as these insects may deposit the pathogens when they feed on food that people consume (27).

We observed a preponderance of isolates of *Proteus* from the slaughterhouse and isolates of *Salmonella* from the eatery. This observation is in line with the findings of Urban and Broce (28) that reported *Proteus* spp. as the most common bacteria among Gram-negative bacteria isolated from flies that were associated with raw meat, followed by *Providencia* spp., *Pseudomonas* spp., and *Salmonella* spp. (29). *Proteus* species are well known as human opportunistic pathogens and intestinal microorganisms indicating fecal pollution of water or soil. Their isolation from flies from the slaughterhouse may be due to the use of such contaminated water for meat processing. In addition, *Proteus* spp. are able to produce volatile components such as putrescine and ammonia, which are important for their swarming ability and, are also able to attract flies to animal carcasses (30). The isolation of *Salmonella* spp from eateries may portend grave danger to public health as this pathogen is associated with gastroenteritis.

Of all the different bacterial species isolated from flies in this study, *S. aureus* was the only gram-positive organism. This organism was isolated from nearly all the locations we sampled demonstrating its ubiquity. Our finding supports previous reports indicating that fly can carry *S. aureus* (25, 31). *Staphylococcus aureus*, an opportunist pathogen, is responsible for a number of human diseases ranging from skin lesions, wound infections and food poisoning to more serious conditions, such as osteomyelitis, endocarditis and septicaemia (32). Therefore, its isolation from the human house and the slaughterhouse portends serious danger to public health.

The results of antibiotic susceptibility tests showed that all isolates were multidrug-resistant. All isolates of *P. aeruginosa*, *Salmonella* spp, and *Proteus* spp were resistant to streptomycin, cotrimoxazole, augmentin, and amoxicillin. Isolates of *E. coli* were commonly resistant to augmentin and cotrimoxazole. The least resistance rates were exhibited by *K. pneumonia*, *E. coli* and *S. aureus* to ciprofloxacin.

Even though, there is a paucity of data on the occurrence of antimicrobial resistance in bacteria associated with flies in Nigeria, independent studies across the globe have nevertheless emphasized the role of flies in the dissemination of resistant bacteria. In a recent global review of the role of flies in the spread of antimicrobial resistance, Onwugambaa et al. (33) revealed that ‘filth flies’ are colonized with clinically relevant antimicrobial resistant bacteria, such as extended-spectrum beta-lactamase, carbapenemase-producing, and colistin-resistant bacteria. In Iran, Davari et al. (5). Isolated cephalexin, chloramphenicol, ampicillin, and tetracycline-resistant bacteria from flies, with resistance against the antibiotics above 32.5%. In a similar study conducted in China, multidrug-resistant enterococci, staphylococci, *E. coli*, *K. pneumoniae*, *P. aeruginosa* and *Aeromonas hydrophila* were identified in flies that were collected beside poultry feeding operations (34, 35). Likewise, Wei et al. showed that antibiotic resistant bacteria can persist in the gut of house and green bottle flies. Animal remains, garbage, hospital waste, and sewage samples are potential sources of resistant bacteria. Hence, flies that are exposed to these sources can easily pick up and disseminate resistant bacteria (36).

Interestingly, some multidrug-resistant bacteria particularly, *Klebsiella*, *Pseudomonas*, and *S.aureus* were isolated from hospital environments. Therefore, hospitals houseflies may also participate more in the spread of antibiotic resistance in the environment. The occurrence of multidrug resistance in clinical isolates is a serious problem due to the waning number of antibiotics used to treat human infections (2, 37). Data from independent studies suggest a link between the antibiotic resistance of food of animal origin, the antibiotic resistance of clinical isolates, and community health (38, 39). Nevertheless, this link remains a contentious issue because of insufficient information on the ecology of antibiotic resistance and virulence genes in the environment (36, 37).

## Conclusion

In conclusion, this present study indicates that house flies (*M. domestica*) are carriers of pathogenic bacteria that pose a possible health risk to communities. The isolated bacteria were resistant to various commonly used antibiotics.

In view of the findings of this study, there is a need for public health education programmes and awareness to be given to the peasants, elites, and patients in the study environment and epidemiological surveillance of food vending joints, major water sources, and hospitals to prevent ingestion of contaminated food and water. Suitable steps should be taken to control the flies and monitor the susceptibility pattern of the pathogens they carry. Further study is needed to determine the type and diversity of all microorganisms spread by flies as well as the epidemiology of the resistant bacteria they carry.
